# Set-up and use of the “Asclepius tube”: a slim drainage tube to facilitate saline-immersion endoscopic submucosal dissection

**DOI:** 10.1016/j.vgie.2025.11.005

**Published:** 2025-11-19

**Authors:** Elisabet Maristany Bosch, Georgios Kalopitas, Elisabetta Dell’Unto, Yoshikazu Hayashi, Hironori Yamamoto, Alberto Murino, Edward J. Despott

**Affiliations:** 1 Royal Free Unit for Endoscopy, The Royal Free Hospital and University College London (UCL) Institute for Liver and Digestive Health, London, UK; 2 Division of Gastroenterology, Department of Medicine, Jichi Medical University, Shimotsuke, Japan; 3 Digestive Disease and Surgery Institute, Cleveland Clinic London, London, UK

## Abstract

**Background and Aims:**

Endoscopic submucosal dissection (ESD) is recognized as the reference standard for en bloc resection of large colorectal and gastric lesions, but conventional carbon dioxide–insufflation ESD presents challenges. The saline-immersion/irrigation technique (SITE) offers multiple advantages, especially when combined with a drainage tube. The video showcases the set-up and practical use of the “Asclepius tube” to optimize SITE-ESD, maintaining its advantages, allowing for a more efficient dissection, keeping an adequate balance of fluid and improving patient comfort.

**Methods:**

A 72-year-old man with a 60-mm sessile lesion (Paris 0-Is) at the rectosigmoid junction was referred for ESD. SITE-ESD was performed using the pocket-creation method with the patient under conscious sedation. The Asclepius tube was wrapped around the scope and connected to continuous low-flow suction (<20 kPa) to drain redundant saline and gas.

**Results:**

The lesion was successfully removed en bloc in 170 minutes (dissection speed of 20.1 mm^2^/min, by 2 fellows training in ESD), without significant intra/postprocedural adverse events. Histopathology confirmed R0 resection of a low-grade dysplastic tubulevillous adenoma.

**Conclusions:**

The Asclepius tube is low cost and easy to set up; it helps maintain optimal fluid balance during SITE-ESD, thus enhancing patient comfort and enabling procedures to be performed with the patient under conscious sedation and without compromising scope maneuverability. Further prospective comparative studies are warranted to validate our findings.

## Introduction

Endoscopic submucosal dissection (ESD) increasingly is recognized as the reference standard for en bloc resection of colorectal and gastric lesions.^1^ In conventional ESD, carbon dioxide insufflation typically is used. However, the introduction of the saline-immersion/irrigation technique (SITE) in 2017^2^ addresses both the drawbacks of gaseous insufflation and previously described underwater ESD techniques with the use of deionized water.^3^ SITE replaces carbon dioxide and water with isotonic saline solution, allowing enhanced optical clarity and magnification; minimization of any gas/liquid interface and lens-soiling with fat; buoyancy of the submucosal flap; improved conductivity through the electrolytes in saline, facilitating vessel precoagulation, and effectively managing intraprocedural bleeding. Our preliminary experience suggests that SITE also enhances patient comfort, because saline, particularly when combined with external drainage, causes less distension than gas.

**Figure 1:**
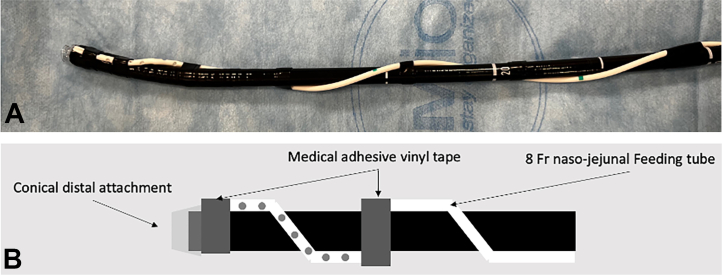
Photograph **(A)** and graphical representation **(B)** show the setting of an 8F nasojejunal fenestrated feeding tube wrapped around the distal part of the endoscope and secured with generic medical adhesive vinyl tape. A conical distal attachment has been previously placed and secured with the same adhesive vinyl tape at the tip of the scope to avoid movement during the procedure.

**Figure 2:**
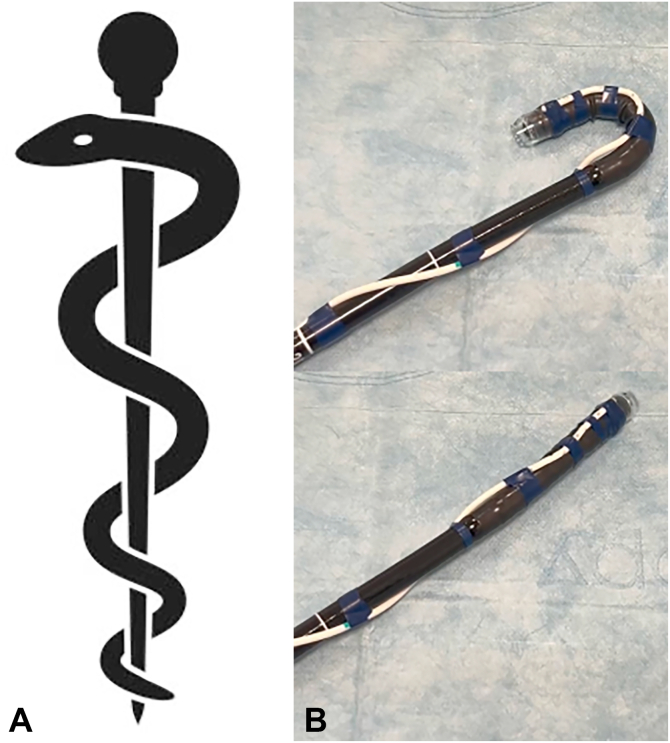
Graphic symbol of “the rod of Asclepius” **(A)**, and photograph **(B)** of the maintained flexibility of the scope with the Asclepius tube wrapped around.

Multiple types of drainage tubes have been described, such as the Foley catheter or transanal tube through gravity-assisted outflow or connected to low-pressure suction, to facilitate distal colonic ESD.^4^^,^^5^ We have also described the use of a slim fenestrated feeding tube wrapped around the scope and secured with vinyl tape to facilitate ESD in the right-sided colon^6^ when using SITE and maintain its advantages. This tube, which we named the “Asclepius tube,” given its similarity to the medical symbol of the snake around the rod of Asclepius^7^ (Figs. 1 and 2), has the objective to drain redundant fluid from the continuous saline-irrigation during ESD and gas generated by diathermy, obviating the need for endoscopic aspiration through the scope. This allows for a more efficient dissection, keeping an adequate balance of fluid inside the lumen, avoiding overdistension of the bowel, maintaining patient comfort, as well as providing better access to the submucosal layer. A retrospective study by Kagaya et al^8^ comparing rectal ESD with or without the Foley catheter showed that the presence of drainage notably improved efficiency of rectal ESD performed by novice endoscopists by increasing dissection speed.

**Figure 3:**
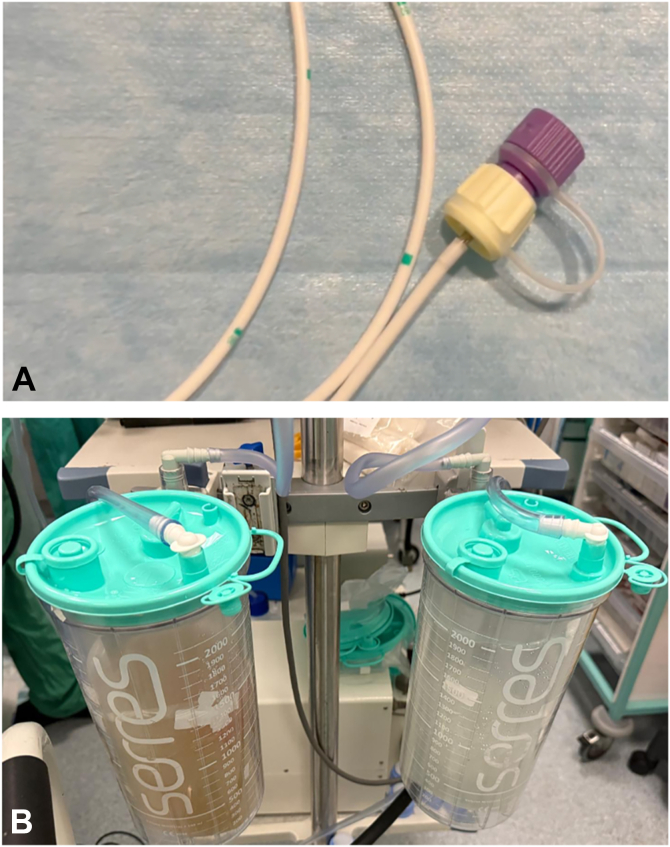
The end tip of the Asclepius tube is connected to the suction attachment **(A)** and attached to continuous low-flow suction (<20 kPa) to drain redundant saline and gas **(B)**.

In our clinical practice, we monitor the saline balance (in vs out) because we find that the optimal working volume of saline to maintain patient comfort and optimize maneuverability for effective and efficient dissection is about 500 mL. We present a video to showcase the straightforward set-up of the Asclepius tube and practical application with ESD of a large rectal lesion performed by 2 novice endoscopists. The case presents a 72-year-old man with a 60-mm sessile (Paris 0-Is, focal Japan Narrow-Band Imaging Expert Team type 2B lesion in the rectosigmoid junction referred for ESD with the patient under operator-delivered conscious sedation (Video 1, available online at www.videogie.org). A slim 7.9-mm gastroscope with a 3.2-mm working channel (EG-840 TP; Fujifilm Healthcare, Tokyo, Japan) was used to achieve maximum maneuverability within the rectosigmoid junction. SITE combined with the bridge-formation variation^9^^,^^10^ of the pocket-creation method^11^, ^12^, ^13^ was used for resection. The scope was equipped with a conical-short, transparent hood (ST Hood DH-083ST; Fujifilm, Tokyo, Japan) to facilitate ESD using the pocket-creation method, and the Asclepius tube (8F nasojejunal tube; Cook Medical Europe, Ltd, Limerick, Ireland) was wrapped around the scope and attached to continuous low-flow suction (<20 kPa) (Fig. 3), initiated at the start of the procedure once the lumen is filled with saline.

A 1.5-mm ball-tipped needle-type knife (FlushKnife BTs 1.5 mm; Fujifilm) was used. Any vessels or mild episodes of bleeding were managed with knife-tip coagulation using swiftCOAG effect 2.5 (VIO 3; Erbe Elektromedizin, Tübingen, Germany) combined with continuous saline irrigation for optimal coagulation effect, causing gradual whitening of the vessel.

**Figure 4:**
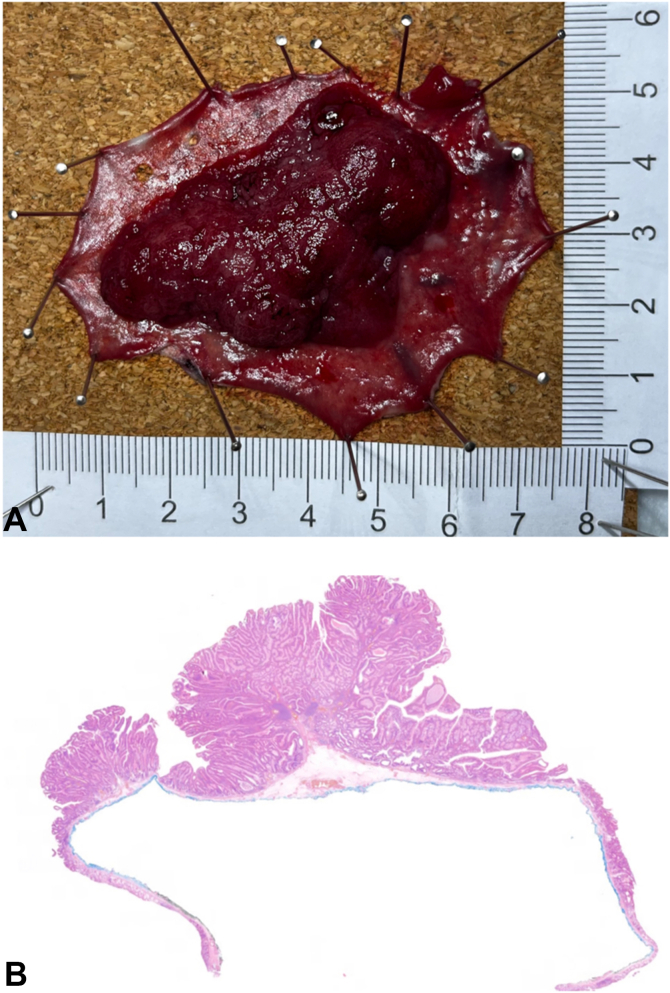
The specimen was removed en bloc **(A)**, and histopathology **(B)** confirmed complete R0, curative resection of a low-grade dysplastic tubulovillous adenoma.

The procedure was completed in 170 minutes (dissection speed of 20.1 mm^2^/min) by 2 fellows training in ESD, without significant intraprocedural adverse events, and the patient was discharged on the same day. Histopathology confirmed R0, curative resection of a low-grade dysplastic tubulovillous adenoma (Fig. 4).

We have started using this tube for all our colorectal SITE-ESDs because it is low-cost and easy to set up and helps maintain an adequate fluid balance during the procedure, enhancing patient comfort, and its use does not appear to affect scope maneuverability or any other hindrance through friction. Further prospective comparative studies are warranted to validate our findings.

## Patient Consent

The patient in this article has given written informed consent to publication of their case details.

## Disclosure

The following authors disclosed financial relationships: H. Yamamoto: Consultant and support (honoraria, grants, and royalties): Fujifilm Corporation. A. Murino: Speaker honoraria: Olympus Medical, Laborie, Boston Scientific, and Fujifilm Healthcare Europe. E. J. Despott: Educational grants in support of conference organization, and honoraria, from Fujifilm Healthcare Europe, Pentax, and Olympus Medical; and honoraria: Ambu and Fujifilm Healthcare Europe. All other authors disclosed no financial relationships.

## Supplementary data

Video 1 Setup and practical use of the Asclepius tube to facilitate the saline-immersion/irrigation technique for endoscopic submucosal dissection of a large rectal lesion.
